# The Inaccuracy of the Mood Disorder Questionnaire for Bipolar Disorder in a Community Sample: From the “DYMERS” Construct Toward a New Instrument for Detecting Vulnerable Conditions

**DOI:** 10.3390/jcm14093017

**Published:** 2025-04-27

**Authors:** Elisa Cantone, Antonio Urban, Giulia Cossu, Michela Atzeni, Pedro José Fragoso Castilla, Shellsyn Giraldo Jaramillo, Mauro Giovanni Carta, Massimo Tusconi

**Affiliations:** 1 Department of Medical Sciences and Public Health, University of Cagliari, Monserrato Blocco I (CA), 09042 Cagliari, Italy; elisa.cantone@libero.it (E.C.); giuliaci@hotmail.com (G.C.); michela.atzeni93@gmail.com (M.A.); maurogcarta@gmail.com (M.G.C.); 2 University Hospital of Cagliari, 09042 Cagliari, Italy; a.urban@aoucagliari.it; 3 PhD Program in Tropical Medicine, Universidad Popular del Cesar, Valledupar 200001, Colombia; pedrofragozo@unicesar.edu.co; 4 Microbiology Program, Universidad Popular del Cesar, Valledupar 200001, Colombia; 5 Faculty of Health, Universidad Popular del Cesar, Valledupar 200001, Colombia; shellsyngiraldo@unicesar.edu.co; 6 Department of Nursing, Universidad Popular del Cesar, Valledupar 200001, Colombia

**Keywords:** Mood Disorder Questionnaire, bipolar disorder, DYMERS, screening accuracy, mental health, social rhythm dysregulation, MDQ, mood disorder

## Abstract

**Background/Objectives:** The Mood Disorder Questionnaire (MDQ) is a widely used tool for the early detection of Bipolar Disorder (BD), yet its diagnostic accuracy remains debated. In particular, the MDQ often yields false positives in individuals with anxiety, stress-related, or personality disorders, raising questions about its clinical utility. This study aimed primarily to evaluate the sensitivity, specificity, and predictive values of the MDQ in identifying BD within a large, community-based sample using structured clinical interviews. Additionally, we explored the construct of DYMERS (Dysregulation of Mood, Energy, and Social Rhythms Syndrome), a proposed condition characterized by mood instability, hyperactivation traits, and rhythm dysregulation among MDQ-positive individuals without a formal psychiatric diagnosis. **Methods:** A total of 4999 adults were surveyed across six Italian regions using a stratified random sampling method. Psychiatric diagnoses were established using DSM-IV-TR criteria via the Advanced Neuropsychiatric Tools and Assessment Schedule (ANTAS). The MDQ was administered face to face in its validated Italian version, with a positivity cut-off of ≥7. The MDQ exhibited low sensitivity and high specificity (0.962; 95% CI: 0.961–0.963). **Results:** Among 2337 analyzable cases, the MDQ showed high specificity (96.2%) but low sensitivity (42.9%) for BD, indicating limited effectiveness as a screening tool. In clinical terms, this implies that while MDQ-positive individuals are unlikely to be false positives, a substantial proportion of true BD cases are not identified. Notably, a significant subgroup of MDQ-positive individuals without psychiatric diagnoses displayed features consistent with DYMERS. **Conclusions:** Our findings confirm the limited screening value of the MDQ for BD in community samples. However, MDQ positivity may help identify a broader spectrum of mood and rhythm dysregulation not captured by current diagnostic systems. Future research should focus on validating DYMERS as a clinical entity and on developing targeted diagnostic instruments capable of capturing this emerging dimension of psychopathology.

## 1. Introduction

Bipolar Disorder (BD) is a chronic and debilitating psychiatric condition associated with high levels of comorbidity, functional impairment, and suicide risk. Despite growing awareness of its burden, BD often remains underdiagnosed or misdiagnosed, which often results in significant delays in appropriate treatment. This diagnostic challenge has led to the development of screening tools aimed at improving early diagnosis in both clinical and community settings. There is a well-known controversy over the use and accuracy of the Mood Disorders Questionnaire and other paper-and-pencil screeners designed to aid in the early diagnosis of Bipolar Disorder [1,2,3,4]. A notable contribution to this discussion comes from a research group at Brown University, which analyzed data from the National Comorbidity Survey Replication. Their findings showed that approximately two-thirds of individuals who screened positive on the MDQ did not meet the criteria for Bipolar Disorder according to structured diagnostic interviews. Instead, a large proportion of these MDQ-positive individuals were found to have anxiety disorders, major depression, or substance use disorders. Furthermore, the study identified elevated levels of psychosocial impairment among MDQ-positive participants, regardless of whether they had a formal psychiatric diagnosis [5,6,7,8]. These results suggest that MDQ positivity may reflect a broader vulnerability to mood and behavioral dysregulation, beyond the categorical diagnosis of Bipolar Disorder, with reverberations on stress, personality, and addiction disorders, even more frequently screened than positives who could be given a clinical diagnosis of Bipolar Disorder [1,2,3,4].

Many psychiatrists with a so-called neo-Kraepelinian [9,10,11,12] approach were led to think that these alleged false positives were the result of a too narrow area in which the official psychiatric nosology relegates Bipolar Disorders [13,14,15,16] and, secondly, of the fact that all the diagnoses found in the so-called false positives were, in reality, conditions frequently found in association with Bipolar Disorder, which notoriously have a later onset [17,18,19,20,21]. The neo-Kraepelinian model, which reasserts the importance of distinct diagnostic categories based on symptom clusters and longitudinal courses, has strongly influenced contemporary psychiatric classification systems. However, emerging models increasingly emphasize the role of transdiagnostic factors such as biological rhythm disruption in the development of mood and stress-related disorders. Alterations in sleep–wake cycles, energy levels, and social routines are now considered core elements not only in Bipolar Disorder but also in broader dysregulatory syndromes. These insights are foundational to the conceptualization of DYMERS, which posits that impairments in rhythmic regulation may constitute an early or alternative manifestation of vulnerability to psychopathology. While the MDQ has demonstrated high specificity in clinical settings, several studies have questioned its broader utility. Much of the critical literature highlights that a substantial proportion of MDQ-positive individuals do not meet diagnostic criteria for Bipolar Disorder when assessed with structured interviews. However, many of these studies rely on samples drawn from clinical populations, which may not reflect the complexity and heterogeneity of the general population. Moreover, most previous research approaches the MDQ within a strictly categorical framework, potentially overlooking subthreshold or atypical patterns of dysregulation. To address these gaps, a series of recent findings, including those from our group, have begun to explore the significance of MDQ positivity in individuals without formal psychiatric diagnoses. These studies suggest that MDQ positivity may reflect a broader vulnerability characterized by mood instability, hyperactivation traits, and social rhythm disruptions, even in the absence of Bipolar Disorder. This has led to the proposal of a new construct—DYMERS—which we aim to explore further in the current study [17].

The Mood Disorder Questionnaire (MDQ) is a widely used self-report screening tool designed to identify individuals who may be at risk of Bipolar Disorder, particularly types I and II. It consists of thirteen yes/no items assessing lifetime experiences of manic or hypomanic symptoms, a question regarding the simultaneous occurrence of these symptoms, and one item on the degree of functional impairment caused. While originally developed for use in clinical settings, the MDQ has also been applied in community-based samples. Its appeal lies in its brevity and ease of administration; however, concerns have been raised regarding its diagnostic accuracy, particularly its sensitivity and its tendency to generate false positives in individuals with other psychiatric conditions.

Some elements produced by our research group have led to a new perspective in the debate [22,23]. First of all, a large group of people with positive results in the MDQ and without a diagnosis of mood disorders [24] or other psychiatric disorders [25] presented a notable impairment in health-related quality of life; this group was also well represented among the elderly who, therefore, did not have a high probability of developing Bipolar Disorders [23,26,27,28,29] or other psychiatric disorders in the future [25,30,31]. Positive results in the MDQ without a diagnosis also presented a substantial dysregulation of social rhythms and sleep in particular, an element characterizing chronic stress [32,33]. Furthermore, it was also found that although the MDQ did not show sufficient accuracy to be used as a screener [34,35,36,37,38], with a certain quota, it was able to identify Bipolar Disorders in a complementary and specular way to an equally imprecise genetic screener [39]. Conversely, among MDQ-positive individuals without psychiatric symptoms, there were also individuals with good social adaptation and high scores on the SF-12 questionnaire [28,40,41,42], which measures perceived quality of life [31,43]. This aspect was observed even though the mean score using this instrument was low across MDQ-positive individuals in a nationwide Italian sample [44]. These findings have led to the hypothesis that MDQ-positive individuals could be categorized into three distinct groups:

1. There are individuals that exhibit novelty-seeking traits and features or episodes of hyper-energy while maintaining overall well-being [45,46,47,48,49]. Many of these individuals exhibit genetic characteristics common to Bipolar Disorder [50]. The prevalence of MDQ positivity and the average MDQ score were higher among Sardinians who had immigrated to Argentina than Sardinians residing in Sardinia [51]. This finding was confirmed in two subsequent comparisons: first, in 2001, during the economic crisis in Argentina, when the frequency of depressive episodes was twice as high among Sardinian-Argentinians; and second, in 2017 [52], during the economic crisis in Italy, when the frequency of depressive episodes was nearly twice as high in a sample of Sardinians residing in Sardinia [53]. Essentially, emigrants, those who had left their homeland, driven by a novelty-seeking impulse, were more prone to experiencing episodes of hyper-energy even in the absence of pathology [54]. Rather than implying a direct link between migration and temperament traits such as novelty-seeking, the observed differences in MDQ scores across Sardinian migrants and residents during periods of socioeconomic crisis may reflect the instrument’s sensitivity to contextual stressors and psychological distress, rather than latent bipolarity or specific syndromic entities. Non-pathological hyper-energy episodes have also been reported among elite athletes before, during, and after their performances [55,56]. Our research also investigates the possible role of DYMERS—a condition characterized by hyperactivation, mood instability, and disruptions in biological and social rhythms—which may act as a subthreshold precursor to mood disorders and may help explain MDQ positivity in individuals who do not meet criteria for Bipolar Disorder.
2. It has been hypothesized that MDQ positivity, even when not associated with a formal diagnosis of Bipolar Disorder, may nevertheless reflect the presence of an underlying condition that has not yet been classified within current psychiatric nosology. This observation, initially made serendipitously, emerged from studies showing that a substantial proportion of MDQ-positive individuals exhibit patterns of hyperactivation, disruption in biological and social rhythms, and diminished perceived quality of life—despite the absence of a diagnosable mental disorder. These findings suggest the existence of a subthreshold or emerging clinical condition, which we propose conceptualizing under the term DYMERS (Dysregulation of Mood, Energy, and Social Rhythms Syndrome) [53,57,58] but is characterized by hyperactivation, the dysregulation of social and biological rhythms, and a low perception of quality of life [24,59]. On the other hand, a certain level of hyperactivation has already been described in the early stages of some stress conditions [60,61]. The syndrome, which is characterized by hyper-energy, the dysregulation of social and biological rhythms, and a low quality of life perception, termed DYMERS, received indirect confirmation during the pandemic [62]. It was observed that mood disorder progression was particularly sensitive to the rhythm dysregulation induced by lockdown measures [63]. Additionally, stress related to rhythm dysregulation mainly affected healthcare personnel, manifesting as stress-related symptoms during the pandemic [64]. The concept that DYMERS could be a relevant construct during pandemic phenomena was subsequently adopted by other research groups [62]. DYMERS might represent a state of nonspecific stress, potentially serving as a starting point for various pathologies, depending on individual vulnerability and the specific nature of the stress-related symptoms experienced [65,66].
3. The next step in evaluating MDQ positivity involves individuals who, after clinical assessment, are diagnosed with Bipolar Disorder. This group is typically less numerous than those with other psychiatric diagnoses but may represent a more severe and clinically complex manifestation of mood dysregulation [67]. According to the literature, which has primarily challenged the accuracy of the MDQ, the prevalence of this condition is lower than that of MDQ-positive individuals with other diagnoses [4,68,69,70,71,72,73]. However, these considerations are primarily based on clinical samples in which the measure of accuracy was affected by an imprecise definition of the prevalence of various disorders [74,75,76,77,78,79], as it would be in the general population, and by the fact that in long-term clinical samples, recall bias is generally more significant than in individuals assessed in the general population [79,80,81,82,83]. In the latter, those with severe psychiatric disorders would be significantly diluted and would not represent the entirety of cases, as seen in clinical samples [84,85]. It is well known that the course of Bipolar Disorder is often characterized by chronic depression that sets in after the “fire” of youthful manic episodes [40,86,87,88,89]. In this condition, while not exclusive yet predominant in long-term depressive disorders, recall bias is likely to be highly significant [89,90,91]. Given the inconsistencies observed in MDQ screening results across various populations, and the unresolved questions regarding its diagnostic utility beyond categorical Bipolar Disorder, the present study aims to assess the MDQ’s effectiveness in identifying BD within a large, representative community sample. Furthermore, we explore whether MDQ positivity may signal a broader, clinically relevant dimension of dysregulation—provisionally conceptualized as DYMERS—among individuals without formal psychiatric diagnoses.

The primary objective of this study is to evaluate the diagnostic accuracy of the Mood Disorder Questionnaire (MDQ) in identifying Bipolar Disorder (BD) within a large, community-based sample. Specifically, we aim to assess the sensitivity, specificity, and predictive values of the MDQ using structured clinical interviews as the reference standard. The secondary, exploratory objective is to investigate the clinical, psychosocial, and functional characteristics of MDQ-positive individuals who do not meet diagnostic criteria for any formal psychiatric disorder. This subgroup is examined with particular attention to the possible emergence of a distinct phenotype characterized by mood instability, hyperactivation, disrupted biological and social rhythms, and impaired perceived quality of life. We propose conceptualizing this syndrome under the provisional term DYMERS (Dysregulation of Mood, Energy, and Social Rhythms Syndrome). Based on these premises, the present study addresses two main research questions: first, evaluating the diagnostic accuracy of the MDQ in identifying Bipolar Disorder in a general population sample; and second, exploring the clinical and functional profile of MDQ-positive individuals without formal psychiatric diagnoses. We hypothesized that while the MDQ would demonstrate high specificity, its sensitivity would be limited in non-clinical settings, and that a distinct pattern of dysregulation—consistent with the proposed DYMERS construct—would be identifiable among undiagnosed MDQ-positive individuals.

The purpose of this study is to verify the MDQ’s effective discriminating capacity in a representative sample of a national community regarding psychiatric diagnosis conducted by clinicians using a semi-structured instrument as the gold standard. This work may better clarify the boundaries of areas of discomfort/distress (not attributable to Bipolar Disorder) that the instrument seems to identify. This study focuses on the Mood Disorder Questionnaire (MDQ), a screening instrument originally designed to identify Bipolar Disorder, and critically evaluates both its diagnostic performance and its potential to detect broader patterns of psychological vulnerability beyond categorical diagnoses.

## 2. Materials and Methods

### 2.1. Design: Epidemiological Community Surveys

We conducted a cross-sectional, community-based survey across six Italian regions using a stratified random sampling method. The design was chosen to obtain a large and demographically diverse sample representative of the general adult population. This approach allowed for the evaluation of the MDQ’s diagnostic accuracy in a real-world, non-clinical setting, thus increasing the ecological validity and generalizability of the findings.

### 2.2. Recruitment and Study Sample

This study’s database was built by interviewing people randomized after stratification by blocks (by age and sex) from six different Italian regions (geographically and economically balanced to be representative of the nation) in the adult population of selected rural and urban municipality records. A detailed description of the survey’s design, recruitment, and conduction has already been published [67]. This study was a community survey. Participants were selected using a proportional stratified random sampling method based on age and sex, in accordance with demographic data from the general population in each of the six Italian regions. This sampling strategy was employed to ensure representativeness and to enhance the external validity of the study results. Face-to-face interviews were carried out at the candidates’ homes. The study sample was randomly drawn from municipal records of the adult population in six different areas, including different Italian locations with varied socioeconomic conditions. These included Sicily, Sardinia, Puglia in the south, Abruzzo in central Italy and Tuscany, and Friuli-Venezia Giulia in northern Italy. In each area, both an urban and a rural sub-area were selected. The urban sub-areas were Iglesias in Sulcis (Sardinia), Catania in Sicily, Bari in Puglia, Pisa in Tuscany, and Udine in Friuli-Venezia Giulia. A third of the sample in each center was drawn from three variously populated municipalities: less than 2000, from 2001 to 10,000, and from 10,001 to 20,000 inhabitants. The sample of Udine was only urban, and Florence was rural.

Randomization was performed after stratification by sex and four different age groups (18–24; 25–44; 45–64; ≥64 years). Using the above-mentioned methodology, a sample of 4999 people was drawn from the seven centers. The size of the sub-drawn samples was 704 in L’Aquila, 971 in Bari, 666 in Catania, 846 in Florence, 465 in Sulcis, 464 in Pisa, and 882 in Udine. General practitioners (GPs) were selected based on geographic location and prior collaboration in epidemiological research projects. They were part of established research networks and volunteered to participate without financial compensation. Their role consisted of identifying eligible individuals from their patient lists and facilitating initial contact for survey participation, thereby enhancing recruitment efficiency and community trust. Each person’s sample included their GP’s name, which was obtained from the general practitioner’s health authority registry (practically every Italian resident is registered with a GP). The relevant GPs were asked to sign an invitation to their patients for survey collaboration. Subjects were contacted at home by phone and by mail by the local coordinator of this study [67]. The six regions included in the study were strategically selected to ensure geographic and socioeconomic diversity, encompassing both urban and rural areas across northern, central, and southern Italy. This approach was intended to maximize representativeness and reflect the heterogeneity of the national population. Participants were excluded from the analysis only if essential diagnostic data were missing—namely, if the ANTAS interview was incomplete or if MDQ responses were absent. Missing demographic data alone did not lead to exclusion unless such variables were required for stratified analyses. This approach was adopted to maintain analytical integrity while minimizing unnecessary data loss.

### 2.3. Study Tools

Demographic records were assessed on an ad hoc basis.

The DSM-IV-TR psychiatric diagnosis was conducted by trained clinical professionals (medical doctors or clinical psychologists with experience working in mental health) by the Advanced Neuropsychiatric Tools and Assessment Schedule (ANTAS), a previously validated semi-structured clinical interview with high reliability in comparison with other diagnostic tools [92,93,94,95]. The ANTAS is a semi-structured clinical interview derived from the SCID (Structured Clinical Interview for DSM-IV), adapted for use in Italian epidemiological studies. It enables standardized psychiatric diagnoses according to DSM-IV-TR criteria. The ANTAS has demonstrated good inter-rater reliability (κ > 0.80) and validity against gold-standard clinical interviews in previous studies.

The Mood Disorder Questionnaire (MDQ) as the Italian version was used to detect lifetime mania or hypomanic episodes but, in the new concept, also episodes of hyperactivity/hyper-energy not attributable to a diagnosis of Bipolar Disorder [96,97,98,99]. The Mood Disorder Questionnaire (MDQ) is one of the most widely used screening tools for Bipolar Disorder. Its primary strengths lie in its brevity, ease of administration, and accessibility across various clinical and non-clinical settings. The MDQ was initially validated with promising sensitivity and specificity values, particularly for Bipolar I Disorder, and has since been translated and adapted into multiple languages. Its structure, based on core features of manic or hypomanic episodes, has contributed to its popularity in primary care and epidemiological studies, where quick screening tools are highly valued. The MDQ is a 13-item self-report instrument designed to screen for lifetime history of manic or hypomanic symptoms. Each item is scored dichotomously (yes/no), followed by two additional questions assessing whether symptoms occur simultaneously and whether they cause moderate or serious problems. A positive screen is typically defined by at least 7 positive symptoms that co-occur and cause moderate to severe impairment. The MDQ has shown high specificity but variable sensitivity across settings. The Italian version has demonstrated adequate internal consistency (Cronbach’s α = 0.79) and test–retest reliability (κ = 0.64)

Health-related quality of life (H-Qol) was measured by the Short-Form Health Survey 12 (SF-12), the scores of which were calculated as described previously in the original publication [100]. The Medical Outcomes Trust (MOT) routinely grants permission to use and reproduce the SF-12 without charge [101,102,103]. The SF-12 is a widely used instrument assessing perceived health-related quality of life across two domains: physical (PCS) and mental (MCS) functioning. It consists of 12 items derived from the original SF-36 and is scored using a norm-based algorithm that yields two composite scores. The SF-12 has demonstrated strong internal consistency (Cronbach’s α > 0.80), test–retest reliability, and validity across various populations, including its Italian adaptation

### 2.4. Ethics

The protocol for the Italian community survey, approved by the ethical committee of the Italian National Health Institute (Istituto Superiore di Sanità) (Rome, Italy), started on 1 August 2006; the approval involved verifying the validity of the instruments used based on the results of the research sample. Informed consent was signed by each interviewee before the interview. This study was conducted in accordance with the ethical standards of the Declaration of Helsinki and approved by the local ethics committees in each of the participating regions. Written informed consent was obtained from all participants prior to their inclusion in the survey.

### 2.5. Statistical Analysis

This study measured the accuracy values of the MDQ at the cut-off of 7 [1], concerning all diagnoses of anxiety or mood disorders. The gold standard was a psychiatric diagnosis according to DSM IV-TR (APA) [104] conducted by clinicians (physicians or psychologists with experience in mental health) adequately trained in the use of a semi-structured interview. Psychiatric diagnoses were based on DSM-IV-TR criteria, in line with the version available at the time of data collection (2005–2006). The ANTAS interview used in this study was calibrated to DSM-IV-TR and allowed for standardized diagnostic assessment. While newer versions of the DSM have since been published, we retained the original diagnostic framework to maintain internal consistency and ensure methodological validity. The sensitivity, specificity, and predictive value of positives and negatives were measured for each diagnosis.

The sample size was not based on a priori power analysis for this specific study but was derived from a larger multicenter epidemiological framework. The total sample of 4999 participants was proportionally distributed across six Italian regions based on population size. This number was considered sufficient to estimate prevalence rates and evaluate diagnostic performance indicators with acceptable precision and statistical confidence. It is important to note that the confidence interval for the sensitivity estimate was relatively wide (0.111–0.796), reflecting the low number of participants diagnosed with Bipolar Disorder in the sample. This limited case count constrains the statistical precision of the sensitivity measure and underscores the need for caution in interpreting this result.

Comparisons of numerical measures were conducted using one-way ANOVA, and comparisons of nominal variables were conducted using chi-square tests.

People with a high level of response to SF-12 in the sample of MDQ-positive individuals without diagnosis were identified as those with a score higher than the mean score of the national normative sample [67] plus one standard deviation or >42.

## 3. Results

Records of a total of 2337 individuals were used for this analysis, of which 1005 were male (43.01%), 1332 were female (56.99%); 998 were <45 years old (42.7%) and 1349 were >44 years old (57.3%). In addition to clinical assessments, detailed demographic and socioeconomic information was collected for all participants. Variables included age, sex, marital status, educational level (primary, secondary, or university degree), and employment status (employed, unemployed, student, retired, or other). These variables were obtained through structured interviews conducted by trained personnel. Regional distribution (north, center, south of Italy) was also recorded to account for geographic variability. This information was used both descriptively and analytically to explore potential associations with MDQ scores and psychiatric diagnoses. Sociodemographic variables, such as younger age and female sex, were significantly associated with higher rates of MDQ positivity. Although educational level and employment status did not show statistically significant effects on diagnostic outcomes, they were considered in the interpretation of quality-of-life differences observed through SF-12 scores.

As shown in Table 1, MDQ-positive individuals were significantly younger and more likely to be female compared to MDQ-negative participants. Additionally, their mean SF-12 scores were substantially lower, indicating poorer self-perceived health. These differences were statistically significant based on chi-square and t-tests (all *p* < 0.001). Table 1 shows the accuracy of the MDQ as a screener concerning mood and anxiety DSM-IV diagnosis; it emerges that only for the diagnosis of Bipolar Disorder did the MDQ show a level of sensitivity apparently almost acceptable for use in two-phase studies, i.e., 0.429 (CI95%: 0.111–0.796), also considering the high specificity (0.962; CI95%: 0.961–0.963) that was similar for all the diagnoses evaluated (from 0.961 to 0.997). However, the predictive value of positives was very low (0.033; CI95%: 0.009–0.081), indicating that the screener did not identify excessive cases of Bipolar Disorder. The positive predictive values for diagnoses such as Panic Disorder (0.120; 95% CI: 0.065–0.199), Obsessive–Compulsive Disorder (0.222; 95% CI: 0.119–0.365), and Major Depressive Disorder (0.098; 95% CI: 0.049–0.127) were higher than that for Bipolar Disorder. However, these values remained too low to support the MDQ as a valid standalone screening tool for any of these conditions. Although OCD and PTSD are no longer classified as anxiety disorders in DSM-5, we report them in this group for consistency with the DSM-IV-TR criteria used in the original diagnostic framework of this study. The positive predictive value was very low (PPV = 0.033; 95% CI: 0.009–0.081), indicating that only a small proportion of individuals with a positive MDQ result actually met diagnostic criteria for Bipolar Disorder. This result is statistically significant and suggests that, despite the MDQ’s high specificity, its reliability as a screening tool is limited in community-based settings due to a high rate of false positives. For Bipolar Disorder, sensitivity was moderate (42.9%), specificity was high (96.2%), the positive predictive value was very low (3.3%), and the negative predictive value was extremely high (99.8%). For Major Depressive Disorder (MDD), sensitivity was very low (8.0%), specificity was high (96.3%), the positive predictive value was low (9.8%), and the negative predictive value was high (95.4%). For Dysthymic Disorder, sensitivity was absent (0.0%), specificity was very high (99.7%), the positive predictive value was negligible (0.0%), and the negative predictive value was high (96.2%). For Panic Disorder, sensitivity was low (12.4%), specificity was high (96.4%), the positive predictive value was low (12.0%), and the negative predictive value was high (96.7%). For Obsessive–Compulsive Disorder (OCD), sensitivity was low (22.2%), specificity was high (96.1%), the positive predictive value was low (10.9%), and the negative predictive value was very high (98.4%). For Post-Traumatic Stress Disorder (PTSD), sensitivity was very low (11.8%), specificity was high (96.1%), the positive predictive value was very low (2.2%), and the negative predictive value was extremely high (99.3%). For Generalized Anxiety Disorder (GAD), sensitivity was very low (5.5%), specificity was high (96.1%), the positive predictive value was very low (3.3%), and the negative predictive value was high (97.7%). Summarizing, MDQ-positive individuals were, on average, younger than MDQ-negative participants and had significantly lower SF-12 scores, suggesting greater perceived functional impairment.

Table 2 shows people with at least one DSM-IV-TR mood and anxiety diagnosis [104] in the overall sample subdivided by positivity in the MDQ and then compared by age, sex, SF-12 score, and the distribution of specific diagnosis. Positive individuals appear younger (40.77 ± 13.51 vs. 47.34 ± 16.36; F = 4.647; *p* = 0.032). The distribution by sex is homogeneous between the two samples, as is the level of H-Qol. Only Bipolar Disorder, among the various diagnoses, presents a higher frequency in the MDQ-positive sample (9.6% vs. 1.4%; chi-square with Yates correction = 20.029; *p* < 0.0001); even if less than 50% of the Bipolar Disorder diagnoses were associated with MDQ positivity (42.9%), all the other diagnoses did not show statistically significant differences in the two groups. However, Agoraphobia presented a discrete tendency to a higher frequency among the negative sample (0 vs. 18.6%; chi-square with Yates correction = 2.099; *p* = 0.148), and OCD had a discrete tendency to a higher frequency among the positive sample (22.6% vs. 12.7%; chi-square with Yates correction = 1.504; *p* = 0.130). To summarize, a strong association was observed between MDQ positivity and the presence of any psychiatric diagnosis, particularly mood and anxiety disorders.

Table 3 shows the individuals without at least one DSM-IV diagnosis in the overall sample divided by MDQ positivity and then compared by age, sex, and SF-12 score. Positive individuals were different from negative ones in mean age (43.45 ± 13.87 vs. 47.25 00B1 14.26; F = 3.939; *p* < 0.049); distribution by sex (females 36.8% vs. 54.7%; chi-square = 14.201; *p* < 0.0001); and H-Qol level (37.05 ± 5.79 vs. 38.90 ± 5.8; F = 55.897; *p* < 0.0001). Despite the low average level of H-Qol among MDQ-positive individuals without a psychiatric diagnosis, in this sample, a frequency of people with a score higher than the mean plus one standard deviation of the national normative sample equal to 21.01% was found; also, due to the large standard deviation, that in the small sample of positive individuals without a diagnosis was comparable to that of the large sample of negative individuals without a diagnosis (5.59 vs. 5.80). Although the MDQ demonstrated high specificity for Bipolar Disorder (96.2%), its positive predictive value was very low (3.3%), indicating that the majority of individuals with a positive MDQ result did not actually meet diagnostic criteria. This finding suggests that, despite its specificity, the MDQ may have limited utility as a standalone screening tool for Bipolar Disorder in general population settings. To summarize, even among participants without any formal psychiatric diagnosis, those who screened positive on the MDQ reported lower quality-of-life scores, indicating that MDQ positivity may reflect broader psychosocial distress.

## 4. Discussion

This study confirms the uselessness of the MDQ as a screener for Bipolar Disorder, even with the result of standardization conducted in a large sample of the community and with the gold standard of psychiatric diagnosis conducted by clinicians through a semi-structured interview [105,106,107,108]. However, contrary to what has emerged from studies on clinical samples, the accuracy of the screener for Bipolar Disorder [85,109,110,111,112] is better than that for other diagnoses of mood and anxiety disorders, including PTSD and OCD [113,114,115,116,117], which in DSM-IV-TR were still included among anxiety disorders [118]. The finding that more than half of individuals diagnosed with Bipolar Disorder tested negative on the MDQ has critical clinical implications. It highlights the tool’s limited sensitivity and suggests that negative MDQ results cannot reliably exclude the presence of BD. Clinicians should therefore exercise caution when interpreting MDQ scores and avoid using the questionnaire as a standalone diagnostic filter, particularly in cases with complex or ambiguous symptom presentations.

This is evident not only as a sensitivity value but, above all, with the result that only Bipolar Disorder, among all the anxiety and mood DSM-IV-TR diagnoses, ended up having a higher frequency [117,119,120,121,122,123,124] in the sample of MDQ-positive individuals compared to the frequency of Bipolar Disorder in the negative sample. However, more than half of people diagnosed with Bipolar Disorder tested negative. This result, in addition to confirming the already established uselessness of the MDQ as a screener [125,126,127], indicates, also based on previous studies, that two different types of Bipolar Disorders probably respond differently to the MDQ. A recent study in older adults found that a genetic test used as a screener, namely the presence of the variant RS1006737 of CACNA1C, was found to be strongly linked to Bipolar Disorder [128,129,130,131]. The MDQ was reasonably accurate in detecting people with a diagnosis of Bipolar Disorder but [131,132,133,134], unfortunately, not accurate enough to be used as a screener for such a disorder [24]. Validity was somewhat mirror-imaged: subjects with a positive genetic test were highly likely to have the disorder, and subjects with a negative MDQ result were highly likely not to have the disorder [134,135], but the reproducibility of the two tests was low, meaning that those who tested positive on one test were not necessarily positive on the other [24]. The current study confirms that there is a high proportion of people with Bipolar Disorder [136,137,138,139] who test negative on the MDQ in the general population, accounting for over 50% of those diagnosed [140,141,142]. These findings are in line with previous studies that have highlighted the MDQ’s limited diagnostic accuracy and have proposed the use of genetic screening or biomarker-based approaches to improve the early identification of Bipolar Disorder. The high rate of false negatives observed in our sample further underscores the limitations of symptom-based screening instruments and reinforces the call for integrated diagnostic strategies that combine clinical, functional, and biological dimensions. The previous above-cited study showed that positives in the MDQ differed in a large proportion to the positives detected by a variant found highly associated with Bipolar Disorder [143,144,145,146] but not present in all people with Bipolar Disorder [147,148,149,150]; this suggested heterogeneity, also from a genetic perspective, between people with Bipolar Disorder who tested positive or negative on the MDQ [73,151].

Although some MDQ-positive individuals without formal diagnoses reported quality-of-life scores at the extreme ends of the distribution, this observation alone does not imply the existence of two distinct populations. Future studies should examine the full distribution of SF-12 scores, including tests for bimodality and comparisons with national norms, before drawing such conclusions.

The genetic heterogeneity observed among individuals with Bipolar Disorder may partly account for the divergent outcomes seen with MDQ screening. Some genetic subtypes may present with less pronounced hypomanic symptoms or different patterns of mood dysregulation that are not adequately captured by the MDQ’s structure. This variability suggests that the MDQ may preferentially detect specific phenotypic or genetic expressions of BD, leaving others underdiagnosed. Such findings highlight the importance of integrating genetic insights into the development of future diagnostic instruments.

It was also found that there was a lack of homogeneity between people with a psychiatric diagnosis of anxiety and depression when dividing them into positive and negative ones in the MDQ. Positive individuals in the MDQ are, in fact, younger [152,153]. Considering that diagnosis on the MDQ is carried out considering lifetimes, this difference may depend (even if not exclusively) on one of the following factors:

(1) A cohort effect with the youngest at risk. This is about a supposed increase in the disorder over time according to the habit’s changes in the new era [12,154]. These habits may have changed the frequency of Bipolar Disorders due to the emergence of a 24 h hectic lifestyle, without neglecting the consequences of light pollution on biological rhythms and melatonin and neurosteroid secretion [155,156]. The higher rate of MDQ positivity observed among younger individuals may reflect a cohort effect driven by contemporary lifestyle and psychosocial factors. Younger generations are increasingly exposed to chronic stress, social instability, digital overstimulation, and irregular sleep–wake cycles, all of which may contribute to emotional and behavioral dysregulation. These influences could amplify subclinical manifestations of mood and rhythm instability, resulting in higher MDQ scores. This pattern supports the hypothesis that MDQ positivity may capture not only latent bipolarity but also broader dysregulatory phenomena consistent with the proposed DYMERS construct.

(2) The effect of recall bias [157]. This is accentuated by the aforementioned condition of frequent chronic depression in older adults diagnosed with Bipolar Disorder and previous mania [40].

(3) An excess of early mortality in individuals positive on the MDQ among whom the frequency of Bipolar Disorder is higher [158,159].

This study also confirms that individuals positive on the MDQ, even in the absence of psychiatric diagnoses, present a low level of perceived H-Qol. These people are also characterized by a higher frequency of women and a younger age, compared to people equally without psychiatric diagnoses but negative in the MDQ, which makes them similar in profile to people who receive a diagnosis. Moreover, it outlines an area of suffering mainly due to the low SF-12 score. However, there is the contradictory aspect of the presence of over a fifth of individuals with a level of H-Qol perception so high as to exceed the mean plus one standard deviation of the national normative data. In conclusion, among those positive in the MDQ without a psychiatric diagnosis, two distinct populations emerge, one of which could be identified as with DYMERS. In contrast, the second is associated with the previously described adaptive hyperactivity. While the SF-12 score difference between undiagnosed MDQ-positive and MDQ-negative individuals is statistically significant, its modest size limits its clinical impact. This result should be interpreted as an exploratory signal rather than conclusive evidence in support of the DYMERS hypothesis.

The MDQ, however, is not a specifically identified tool for recognizing DYMERS. Therefore, a specific questionnaire should be constructed that, in addition to hyperactivity, could measure stress and rhythm dysregulation.

This study has several limitations that should be acknowledged. First, the data were collected in 2005–2006, and psychiatric diagnoses were based on DSM-IV-TR criteria, which have since been updated in DSM-5 and DSM-5-TR. While the diagnostic framework remains relevant, some classification changes may limit comparability with more recent studies. Second, the MDQ is a self-report instrument, which may be subject to recall bias or the misinterpretation of symptomatology, especially in a community-based sample. Third, the cross-sectional design precludes conclusions about causality or the longitudinal trajectory of MDQ-positive individuals, including those without a formal diagnosis. This study highlights the limited clinical utility of the MDQ as a screening tool for Bipolar Disorder in community samples, while suggesting its potential to detect broader patterns of emotional and behavioral dysregulation. These findings support the need for future research into transdiagnostic constructs such as DYMERS and the development of more refined, multidimensional screening instruments.

The findings suggest that the MDQ, while highly specific, has limited sensitivity and a low positive predictive value for Bipolar Disorder in non-clinical populations. This significantly undermines its utility as a standalone screening tool in community or primary care settings. However, the association between MDQ positivity and patterns of dysregulation among individuals without formal psychiatric diagnoses raises important questions about subthreshold psychopathology. Future research should aim to longitudinally track MDQ-positive individuals, explore the biological and psychosocial underpinnings of the DYMERS construct, and develop multidimensional screening instruments that better capture early or atypical manifestations of mood and rhythm dysregulation.

Future studies should consider using receiver operating characteristic (ROC) curve analysis to explore alternative MDQ cut-off scores that may better balance sensitivity and specificity in general population samples, as the standard threshold of ≥7 symptoms may not be optimal in low-prevalence contexts.

In summary, this study highlights the limitations of the Mood Disorder Questionnaire as a diagnostic screener for Bipolar Disorder in community samples, given its low sensitivity and poor predictive value. Nonetheless, the consistent association between MDQ positivity and psychosocial dysfunction—particularly among individuals without psychiatric diagnoses—points to the existence of a broader, understudied clinical dimension. The proposed DYMERS construct may offer a useful conceptual framework for capturing this phenomenon. Further validation and operationalization of this syndrome are necessary to inform future diagnostic systems and early intervention strategies.

## Figures and Tables

**Table 1:** Accuracy of MDQ as screener concerning mood and anxiety DSM-IV diagnosis.

Type	Estimated Prevalence %	Sensitivity	95% CI	Specificity	95% CI	PPV	95% CI	PNV	95% CI
Bipolar Disorder	0.3	0.429	0.111–0.796	0.962	0.961–0.963	0.033	0.009–0.081	0.998	0.997–0.999
MDD	4.8	0.080	0.040–0.143	0.963	0.961–0.966	0.098	0.049–0.127	0.954	0.952–0.957
Dysthymic Disorder	0.04	0.000	0.000–0.035	0.997	0.997–0.998	0.000	0.000–0.435	0.962	0.962–0.964
Panic Disorder	3.6	0.124	0.068–0.206	0.964	0.962–0.967	0.120	0.065–0.199	0.967	0.965–0.970
OCD	1.8	0.222	0.119–0.365	0.961	0.960–0.967	0.109	0.058–0.178	0.984	0.982–0.987
PTSD	0.7	0.118	0.021–0.371	0.961	0.960–0.963	0.022	0.004–0.069	0.993	0.993–0.995
GAD	2.4	0.055	0.014–0.155	0.961	0.960–0.963	0.033	0.009–0.093	0.977	0.976–0.979

**Note:** BD = Bipolar Disorder; MDD = Major Depressive Disorder; Dysthymic Disorder = Persistent Depressive Disorder; Panic Disorder = Panic Disorder; OCD = Obsessive–Compulsive Disorder; PTSD = Post-Traumatic Stress Disorder; GAD = Generalized Anxiety Disorder; PPV = Positive Predictive Value; NPV = Negative Predictive Value; CI = Confidence Interval.

**Table 2:** Prevalence of psychiatric diagnoses among MDQ-positive participants.

Item	MDQ-Positive Individuals with a Diagnosis of DSM-IV-TR Mood and Anxiety Disorders (Including PTSD and OCD) (N = 31)	MDQ-Negative Individuals with a Diagnosis of DSM-IV-TR Mood and Anxiety Disorders (N = 283)	ANOVA 1311 df or χ^2^ 1 df
Age (Years)	40.77 ± 13.51	47.34 ± 16.36	F = 4.647 *p* = 0.032
Female	25 (80.6%)	211 (74.5%)	
SF-12 Score	34.03 ± 5.62	35.39 ± 6.41	F = 1.286 *p* = 0.258
Bipolar Disorder	3 (9.6%)	4 (1.4%)	χ^2^ = 20.029 (Yates correction) *p* < 0.0001
MDD	8 (25.8%)	89 (31.44%)	χ^2^ = 0.417 *p* = 0.519
Dysthymic Disorder	0 (0)	7 (2.5%)	χ^2^ = 0.060 (Yates correction) *p* = 0.807
Panic Disorder	8 (25.8%)	75 (26.5%)	χ^2^ = 0.001 *p* = 0.999
GAD	3 (9.6%)	50 (17.6%)	χ^2^ = 1.271 (Yates Correction) p = 0.529
Agoraphobia	0 (0)	18 (6.4)	χ^2^ = 2.092 (Yates Correction) *p* = 0.148
Specific Phobia	5 (16.1%)	50 (17.6%)	χ^2^ = 0.001 (Yates Correction) *p* = 0.999
Social Phobia	1 (3.2%)	11 (3.9%)	χ^2^ = 0.001 (Yates Correction) *p* = 0.999
PTSD	2 (6.5%)	14 (4.9%)	χ^2^ = 0.001 (Yates Correction) *p* = 0.999
OCD	7 (22.6%)	36 (12.7%)	χ^2^ = 1.540 (Yates Correction) *p* = 0.130

**Note:** Age (Years) = participants’ age in years; Female = gender (female); SF-12 Score = Short-Form Health Survey 12 total score; BD = Bipolar Disorder; MDD = Major Depressive Disorder; Dysthymic Disorder = Persistent Depressive Disorder; Panic Disorder = Panic Disorder; GAD = Generalized Anxiety Disorder; Agoraphobia = Agoraphobia; Specific Phobia = Specific Phobia; Social Phobia = Social Anxiety Disorder; PTSD = Post-Traumatic Stress Disorder; OCD = Obsessive–Compulsive Disorder.

**Table 3:** Functional impairment (SF-12 scores) in MDQ-positive participants without a formal psychiatric diagnosis.

Item	MDQ-Positive Individuals without Diagnosis (N = 57)	MDQ-Negative Individuals without Diagnosis (N = 1966)	ANOVA 12,021 df or χ^2^ 1 df
Age	43.45 ± 13.87	47.25 ± 14.26	F = 3.939 *p* < 0.049
Female	21 (36.8%)	1075 (54.7%)	χ^2^ = 14.201 *p* < 0.0001
SF-12 Mean Score	37.05 ± 5.79	Mean 38.90 ± 5.80	F = 55.897 *p* < 0.0001

**Note:** Age = participants’ age in years; Female = gender (female); SF-12 Mean Score = average score on Short-Form Health Survey 12; MDQ = Mood Disorder Questionnaire. Only participants without DSM-IV-TR psychiatric diagnoses are included in this table.

## Data Availability

The data presented in this study are available upon request from the corresponding author. Due to privacy and ethical issues, data are not publicly available.
